# Trajectory of lung ultrasound scores in preterm infants at risk for bronchopulmonary dysplasia

**DOI:** 10.1038/s41372-026-02561-9

**Published:** 2026-03-13

**Authors:** Rachel M. Weinstein, Cassandra R. Montoya, Russell Horowitz, Karna Murthy, Leslie A. Caldarelli, Stephanie M. Marshall

**Affiliations:** 1 Division of Neonatology; Feinberg School of Medicine, Northwestern University & Ann and Robert H. Lurie Children’s Hospital of Chicago, Chicago, IL USA; 2 Division of Pediatric Emergency Medicine; Feinberg School of Medicine, Northwestern University & Ann and Robert H. Lurie Children’s Hospital of Chicago, Chicago, IL USA

**Keywords:** Prognostic markers, Respiratory tract diseases

## Abstract

**Objective:**

To quantify the trajectory of lung ultrasound scores (LUSSc) and estimate their association on inpatient respiratory trajectories in preterm infants with potentially evolving bronchopulmonary dysplasia (BPD)

**Study design:**

We measured LUSSc prospectively in infants <32 weeks’ gestation weekly from 2 weeks of life through 44 weeks’ post-menstrual age (PMA) or discharge. The primary outcome was the age, in PMA, to achieve liberation of respiratory support (LRS) defined as room air or < 1 L/min (F_i_O_2_ = 100%).

**Result:**

In 16 infants, 10 patients had Grade 2–3 BPD, and LUSSc declined over time. The median age to achieve LRS was 42 weeks’ PMA (33.6, 47.9). Using the maximum LUSSc each infant had between 30 and 32 weeks’ PMA, each 1-point increase in the LUSSc was independently associated with a 9-day increase in the age to achieve LRS.

**Conclusion:**

LUSSc may serve as a diagnostic marker for inpatient respiratory trajectories of preterm infants with evolving BPD.

## Introduction

Bronchopulmonary dysplasia (BPD) is the most frequent morbidity in preterm infants graduating from the neonatal intensive care unit (NICU) [1] and is associated with prolonged mechanical ventilation and hospitalization, cardiac dysfunction, pulmonary hypertension, and pediatric asthma [2]. Since 2005, the severity of BPD has been associated with adverse early childhood neurodevelopmental outcomes [3, 4]; yet, these relationships are based on a definition of BPD founded in the types and amount of respiratory supports—with subjective indications and applications--provided at 36 weeks’ post-menstrual age (PMA) instead of objective, clinical markers of pulmonary disease. How these markers may relate to, and even predict, outcomes for infants at-risk for BPD remain uncertain.

Point of care lung ultrasound (LUS) has emerged as a reproducible, safe, real-time imaging modality [5]. Prior studies have defined LUS as a radiographic biomarker for BPD in the NICU [5–8]. Early lung ultrasound assessments are shown to predict BPD [3, 9–13]. However, serial lung ultrasound assessments are not yet correlated to respiratory support needs (or liberations of said support) in affected infants [4]. Understanding the trajectory of lung parenchymal change and its correlations with practical milestones in neonatal intensive care would be a novel contribution, as few studies focus on how these changes relate to ongoing respiratory needs in infants with evolving BPD [4, 14].

This study aimed to investigate the association between lung ultrasound scores (LUSSc) and time to liberation of respiratory support, room air or <1 L/min (F_i_O_2_ = 100%), in a cohort of preterm infants at risk for BPD. We hypothesize that the LUSSc can be used to risk-stratify infants quantitatively toward more favorable or adverse short-term inpatient trajectories.

## Methods

### Patients

This was a two-center, prospective, observational cohort study conducted in academic tertiary and quaternary level neonatal intensive care units (NICU) at Prentice Women’s Hospital and Lurie Children’s Hospital in Chicago, Illinois. Infants born <32 weeks’ gestation between September 1, 2022, and December 31, 2023, were screened. Infants with chromosomal aneuploidy and critical congenital heart disease (apart from PDA and PFO) were excluded from the study. Data pertaining to the patients’ baseline characteristics, mode and duration of respiratory support, and morbidities associated with prematurity were recorded prospectively until hospital discharge.

The primary outcome was the post-menstrual age (PMA) in weeks required to achieve liberation of respiratory support (LRS), defined as room air or <1 L/min (F_i_O_2_ = 100%). Secondary clinical factors of interest were tracheostomy placement or death prior to 6-months of age, moderate or severe bronchopulmonary dysplasia, treatment of retinopathy of prematurity (laser or anti-vascular endothelial growth factor (VEGF) injections), grade III-IV intraventricular hemorrhage, and pulmonary hypertension at 36 weeks’ PMA as defined by echocardiogram. Echocardiographic determination of pulmonary hypertension was per the attending cardiologist’s discretion and graded as mild, moderate, or severe. BPD was defined as grade 1–3 [15].

### Lung ultrasound protocol

**Fig. 1:**
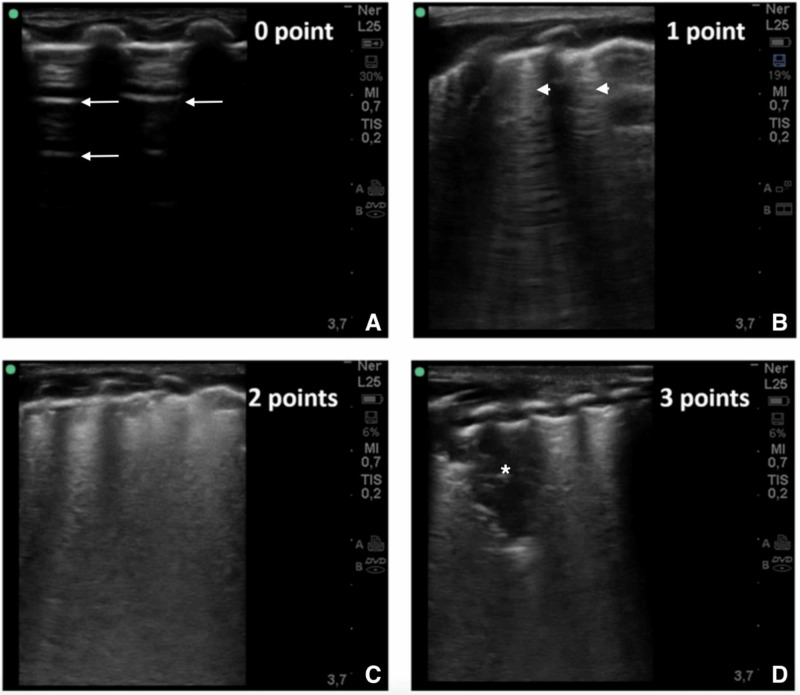
Lung ultrasound scoring and sonographic appearance. **A** 0 points; normally aerated lung with A line pattern (A-line denoted by arrows) that represents reflections of sonographic waves off the pleura in a non-obstructed air-filled lung. **B** 1 point; mild interstitial syndrome with non-confluent B-line pattern (B-line denoted by arrow heads) representing transmission of sonographic waves through lung parenchyma with increased density filling the pulmonary interstitial and/or alveolar spaces. **C** 2 points; severe interstitial syndrome with confluent B-line pattern with or without evidence of consolidation < 1 cm in depth from the pleural line. **D** 3 points; extensive lung consolidation (Denoted by asterisk) with coalescent B-lines with sub-pleural consolidation ≥ 1 cm in depth from the pleural line extending into the parenchyma.

Lung ultrasounds were completed weekly beginning at 2 weeks of age by three trained sonographers (CM, RW, SM). Scans were completed with a commercial transportable ultrasound device (SonoSite SII) using a high frequency linear probe (13–6 MHz). The scanning protocol included five lung zones in each lung (anterior superior, anterior inferior, lateral, posterior superior, and posterior inferior: as shown in Fig. 1 in Zong et al.) [16]. Anterior and lateral zones were assessed in the supine position, while posterior zones were assessed in the lateral decubitus position. Two, 3–5 s cine clips were taken of each lung zone and stored for later analyses. The patient’s position was recorded (supine, prone or lateral decubitus) one hour prior to the scan. Aeration in each lung zone was scored from 0 to 3 points (total score ranging from 0 to 30), using a validated extended lung ultrasound scoring protocol [17]. The sum of the total score serves as an index for the severity of lung disease. The LUSSc was assigned as follows: 0 indicates A-pattern (defined by the presence of only A lines); 1, B-pattern (defined by the presence of ≥ 3 well-spaced B-lines within the frame); 2, severe B pattern (defined as the presence of coalescent B-lines with or without consolidation < 1 cm in depth from the pleural line); and 3, coalescent B-lines with extended sub-pleural consolidation (Fig. 1). Lung ultrasound findings have been shown to correlate closely with extravascular lung water measured by transpulmonary thermodilution [18]. Per multiple prior studies, A-lines represent reflections of the pleural line that are visible when ultrasound waves diffuse through a non-obstructed air-filled lung. B-lines occur when there is increased lung density filling the interstitium and/or alveolar space [16–20].

Lungs scans were scored by two physician reviewers credentialed in point of care ultrasound acquisition and interpretation. One physician reviewer who did not scan these infants was blinded (RH) to clinical course/outcomes; the other reviewer scanned infants, and thus, was aware of infants’ clinical trajectory (SM). The interrater intraclass correlation coefficient was quantified.

### Statistical analysis

Description of LUSSc by PMA was the first goal to quantify how these sonographic markers of pulmonary pathology evolve during the NICU hospitalization. Given the hypothesized potential difference in quantifying the LUSSc, we reported the trajectory of LUSSc as modeled by our two reviewers of each scan. The interrater intraclass correlation coefficient was calculated to compare the agreement between the 2 reviewers’ scores.

Then, to understand the presence and/or magnitude of the relationship between LUSSc and age to achieve LRS, we utilized the maximum LUSSc obtained between 30-32 weeks’ PMA, a full 4 weeks before the diagnosis of BPD is established. We chose this time point after reviewing our descriptive data reported in Fig. 4. This maximum score was used as the main exposure on the outcome of age-at LRS (in weeks PMA). Candidate confounding variables were incorporated as those associated with the exposure and outcome and/or those hypothesized to be related to the severity of BPD (e.g., SGA < 10^th^ centile, sex, position of infant during the scan, and respiratory support during scan).

We conducted quantile regression to examine the association between [exposure variable, e.g., total lung ultrasound score (LUSSc)] and the outcome variable ([Time to LRS, expressed in PMA]) in unadjusted association. Quantile regression estimates conditional quantiles (e.g., the median) of the outcome distribution rather than the mean. We used this approach because of our small sample size and because the outcome distribution of time to LRS is non-parametrically distributed and skewed. We estimated median regression models (τ = 0.5) and reported regression coefficients with 95% confidence intervals. Our limited sample size precluded estimations of quantiles other than the reported median (e.g., 25^th^%ile, 75^th^%ile). Of note, gestational age was not considered in these models as it is embedded in the calculation of age (PMA).

In secondary analyses, to describe the determinants of LUSSc and their inpatient trajectories, we the collected clinical variables (e.g., sex, gestational age, SGA<10^th^ centile, grade 2–3 BPD) and quantified the unadjusted associations between these variables and LUSSc [21]. Those variables that were significantly related to LUSSc (*p* < 0.2) in unadjusted analyses were incorporated into the multivariable linear regression model via backward selection. For these computations, we employed a time-series regression analyses with autocorrelations to examine how each infant’s LUSSc changed over time with respect to their clinical/demographic characteristics. We report the model incorporating clinical characteristics associated with the weekly change in LUSSc.

This study was reviewed by the IRB at Ann & Robert H. Lurie Children’s Hospital of Chicago (IRB 2021-4037). Informed parental/guardian consent was obtained prior to participation. Exams were deferred to a later date if a patient was deemed too clinically unstable to tolerate the exam by the treating medical team. Redcap with two-factor authentication was used for confidential storage of patient clinical characteristics and STATA (v17.1) was used for statistical analyses. All hypothesis tests were 2-sided, and *p* < 0.05 was statistically significant.

## Results

Sixteen infants were enrolled. The mean gestational age at birth was lower for those who developed BPD (No BPD: 30 [IQR = 30–31] weeks’ gestation; BPD: 26 [IQR 24–29] weeks’ gestation). The mean birthweight of infants without BPD was 1570 g (SD 351 g) and 849 g (SD 194) for those with BPD. Most infants were male and peripartum infection such as chorioamnionitis was infrequent (Table 1). Both cesarean delivery and a complete course of antenatal betamethasone were present in most infants (Table 1).

**Table 1:** Characteristics of the study population (*n* = 16). Values expressed as n (%) or median (25^th^–75^th^%ile), as appropriate.

Variable	
Gestational age, weeks	29 (25.5, 30)
Birthweight (g)	1030 (786-1383)
Male sex	11 (68.8)
Small for gestational age <10^th^ centile (Olsen 2010)	3 (18.8)
Maternal Race	
White	8 (50)
Black	6 (37.5)
Other	2 (12.5)
Maternal diabetes	3 (18.8)
Pre-eclampsia	4 (25)
Maternal fever	1 (6.3)
Vaginal delivery	6 (37.5)
Antenatal steroids (2 doses, >48 h prior to delivery)	11 (68.8)
Surfactant administration	9 (56.3)
Postnatal steroids	3 (18.8)

**Fig. 2:**
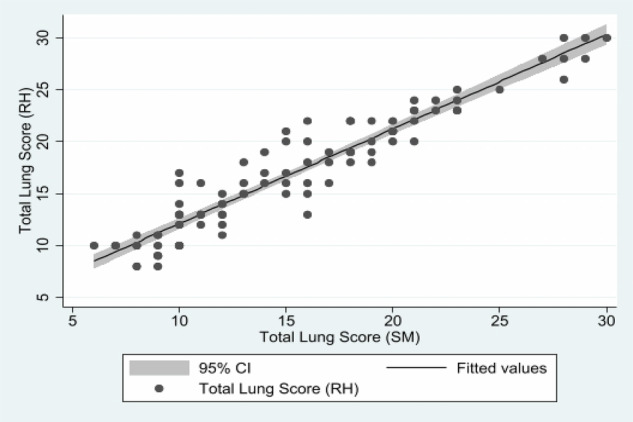
Total lung scores described by two reviewers demonstrating the reliability of LUSSc. Inter-rater intraclass correlation coefficient, 0.73, 95% CU (0.39-1.00, *p* = 0.057) demonstrating modest agreement in scoring. Each image was scored 0-3 as described in Figure 1.

**Fig. 3:**
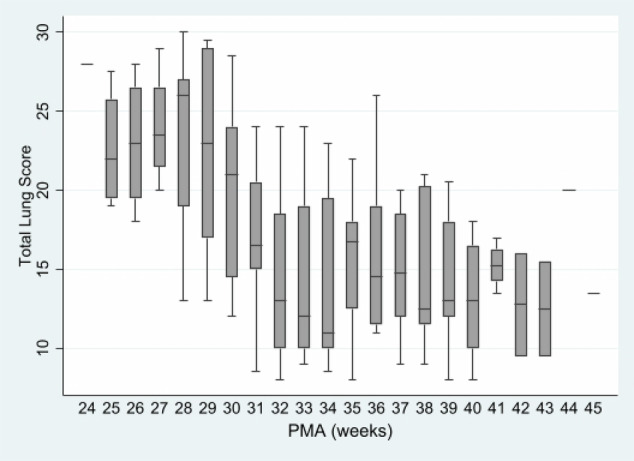
Total Lung Ultrasound Score ((LUSSc), *n* = 16 infants and their 152 images) vs. Post-Menstrual Age (weeks). The median total LUSSc decreases after 30-32 weeks' PMA in these enrolled infants.

**Fig. 4:**
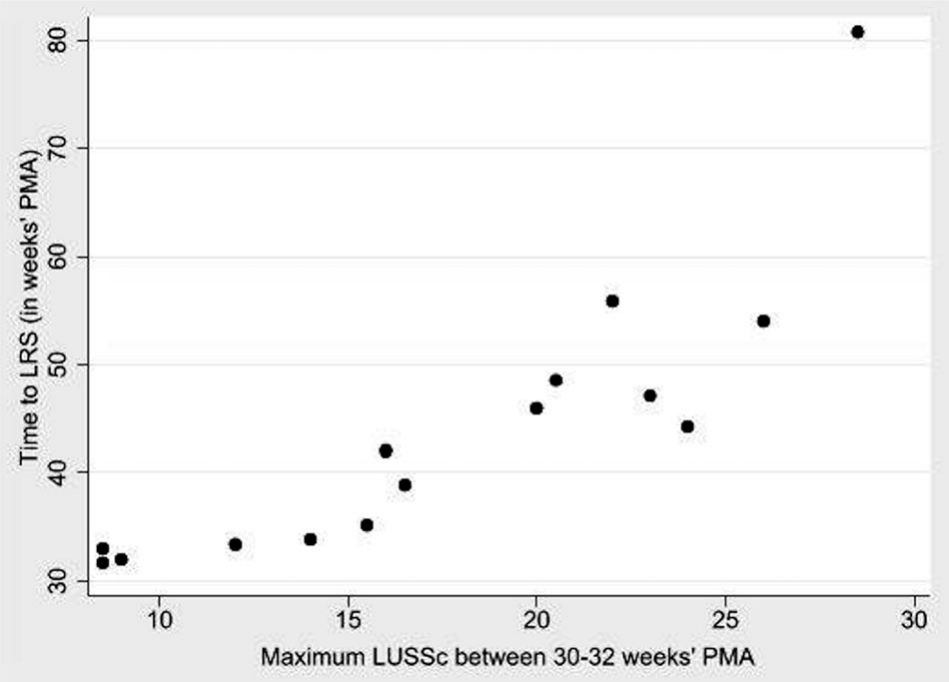
Time to liberation of respiratory support (LRS)  for infants as a function of each infant's maximum LUSSc between 30 and 32 weeks’ PMA. These data report a relationship between these maximum LUSSc and the duration to attain LRS.

One hundred and fifty-two total lung ultrasounds were completed. There was moderate to good reliability of lung ultrasound scores granted between the two physician reviewers (ICC 0.73, 95% CI: 0.39–1.00, *p* = 0.057) (Fig. 2). The age to achieve LRS was related to the maximum LUSSc documented between 30 and 32 weeks’ PMA in unadjusted analyses (Fig. 3) with none of the hypothesized co-variates remaining significantly associated with both LUSSc and the outcome in multivariable analyses. Quantile regression analyses based on the raw data presented (Fig. 4) demonstrated that each one-point increase in the maximum LUSSc at 30–32 weeks’ PMA was associated with a 1.29-week (or ~9 days) increase in duration to reach LRS (*p* < 0.01). Correspondingly, the median predicted (95% CI) time-to-LRS was 41.4 (31.4, 57.2) weeks’ PMA. Gestational age, respiratory support during the scan, position of the infant 1-h prior to the scan (e.g., supine or prone), receipt of corticosteroids, and SGA <10^th^ centile was unrelated to the primary outcome. These negative associations may be subject to type II error.

**Fig. 5:**
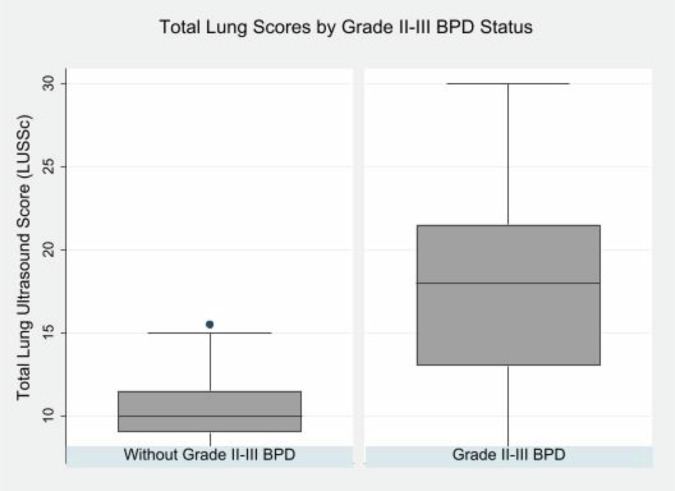
Maximum total LUSSc measured at 30-32 weeks’ PMA in 16 infants stratified by the presence of Grade 2–3 BPD y (Wilcoxon Rank-Sum Test, *p* = 0.04) as per Jensen et al, 2019.

LUSSc were higher among those with grade 2–3 BPD (Fig. 5), (*p* < 0.001, Fig. 5) relative to those without BPD with largest differences noted between 30 and 32 weeks’ PMA (Grade 2–3 BPD: LUSSc of 19, vs no BPD: LUSSc of 11, *p* = 0.04).

To gain insights into the natural history of LUSSc in preterm infants, secondary analyses evaluated whether clinical variables were associated with LUSSc. Multivariable analysis (Table 2) shows LUSSc was related to type of respiratory support, sex, gestational age, and exposure to systemic steroids. Patient position 1 h before scanning demonstrated a trend but this particular variable did not reach statistical significance. To describe the regression model, receipt of any systemic steroids was associated with a 2.98-point decrease per week in LUSSc after adjusting for sex, gestational age, and type of respiratory support. Of note, in univariable analysis, receipt of systemic steroids (*p* = 0.11) or its cumulative dose (median dose 1.52 mg/kg, [25^th^–75^th^ percentile: 0.82–2.27 mg/kg]) was unrelated to the time to LRS with or without adjustments for gestational age.

**Table 2:** Multivariable linear regression analysis to isolate determinants of LUSSc.

Variable	*β*	95% CI	*P*
Patient position	0.57	–0.088, 1.23	0.09
Gestational age (weeks)	–1.87	–0.244, –1.31	<0.01
Sex*	–2.76	–4.80, –0.724	0.01
SGA	4.9	2.34, 7.47	<0.01
Mode of respiratory support^	–1.41	–1.82, –1.01	<0.01
Systemic steroids	–2.98	–5.57, –0.231	0.034

Regression analysis. *Referent group = male. ^ Referent to invasive

mechanical ventilation. SGA, small for gestational age (<10th percentile).

Lastly, nearly 20 percent of infants had mild pulmonary hypertension on echocardiography at 36 weeks’ PMA [15]. Bacteremia and BPD were prevalent as was clinical improvement such that the majority of the cohort was discharged in room-air (Table 3).

**Table 3:** Clinical outcomes of study population (*n* = 16). Values expressed as n (%) or median (25^th^–75^th^%ile), as appropriate.

Bacteremia	3 (18.8)
Non-surgical necrotizing enterocolitis	1 (6.3)
Intraventricular hemorrhage, grade III-IV	3 (18.8)
Bevacizumab therapy for retinopathy	2 (12.5)
Percutaneous ductus arteriosus occlusion	3 (18.8)
Pulmonary hypertension at 36 weeks’ PMA	3 (18.8)
Grade 2–3 bronchopulmonary dysplasia	10 (62.5)
Respiratory support at discharge	
Room air	13 (81.3)
Nasal cannula	2 (12.5)
Tracheostomy with ventilator	1 (6.3)
Liberation of respiratory support (Post-menstrual age in weeks)	42.1 (33.6, 47.9)

## Discussion

In this prospective observational study in infants born <32 weeks’ gestation, LUSSc markers appear to be relatively static until approximately 30–32 weeks’ PMA. These scores appear to decrease, in particular among those who have short-term reductions in respiratory supports, likely related to clinically relevant improvement in their pulmonary disease. Specifically, increases in the LUSSc were related to an increase in duration to achieve LRS. With a moderate inter-rater intraclass correlation, these results suggest that LUSSc and their trajectories can be used to increase precision of parental counseling and diagnostic certainty toward a clinically relevant outcome for inpatients in our NICU. These findings suggest that a fall in LUSSc may be a necessary finding to correlate with clinical improvement during the NICU inpatient stay, and furthermore, therapies that may be targeted to decrease LUSSc in this time window may be an area of future promise to assist infants affected by or at-risk for BPD.

Both classic and extended protocols for the LUSSc, at days 7 and 14 of life, in infants born <32 weeks’ gestation have been shown to predict BPD with good diagnostic accuracy [13, 22]. These results present an exciting opportunity for the increased use of bedside point of care lung ultrasound to personalize care for premature infants with chronic pulmonary insufficiency. To our knowledge, few studies have examined the weekly longitudinal progression of the LUSSc in preterm infants with evolving lung disease.

Alonso-Ojembarrena and colleagues looked at the LUSSc in very low birth weight infants in the first 24- and 72-h of life and then weekly until 36 weeks’ PMA [23]. Our study designs differed in that our primary outcome was PMA to achieve LRS *versus* prediction of BPD using the LUSSc at various timepoints. As such, we did not begin LUS until 2-weeks of life, in order to allow infants to progress from initial respiratory failure from surfactant deficiency to more chronic pulmonary insufficiency [22]. Interestingly our study, which was smaller in size (16 vs 59) but had a larger cohort of infants who developed grade 2–3 BPD (62% vs 38%), showed a distinct reduction in LUSSc at 30-32 weeks’ PMA. More investigation is required to elucidate the pathophysiology driving changes in LUSSc over time.

Our study is the first to imply that an increase in LUSSc following an initial period of improvement may be indicative of a longer time to achieve LRS. Previous studies have shown that the LUSSc inversely correlates with lung aeration and lung volume as well as oxygenation and dyspnea scores [24, 25]. Given the heterogeneity of phenotypes and outcomes for infants with BPD, a changing LUSSc may assist neonatologists in guiding targeted therapy, such as diuretics, steroids or change in respiratory support, for at risk infants as well as provide more detailed counseling to families.

In our study, we used an extended lung scoring protocol, which includes posterior lung fields, as some studies have shown that the posterior lung fields (e.g. gravity-dependent lung zones) in inflammatory diseases, such as BPD and bronchiolitis, are less aerated [9, 12]. Our regression model showed that patient position (supine, prone or side-lying) 1-h prior to lung ultrasound was unrelated to the trajectory of LUSSc over time. These results fall in line with a meta-analysis that reviewed LUSSc of over 1000 neonates, which showed no difference in diagnostic accuracy in predicting BPD between the classic scoring system, which excludes posterior lung fields, and the modified or extended scoring system [22]. These results suggest that inclusion of posterior lung fields may not be necessary to assess the severity of evolving lung disease in preterm infants at risk for BPD, allowing for more efficient and less burdensome lung ultrasound exams at the bedside. Perhaps this is due to the heterogenous pathology of BPD. However, further studies are needed to better characterize lung ultrasound findings in infants with evolving BPD to corroborate this observation.

We acknowledge our study’s limitations including the small sample size and lack of blinding of one of the physician reviewers. Pulmonary hypertension on echocardiogram was determined by the cardiology attending’s individual discretion as there are variations in the clinical grading of pulmonary hypertension by echocardiogram in neonates. Regarding the generalizability of our results, our sample size limits general inferences; however, there is little reason to believe these infants are dissimilar in their presentation or in their LUSSc from a larger cohort in other settings. Indeed, further validation is required to confirm these observed associations. Time to LRS is not uniform as it is subjectively determined by a changing medical team. As with all ultrasound studies, there are inherent limitations of ultrasound that are important to consider, such as an inability to evaluate vasculature, airway disease, cystic lung changes, and pulmonary hypertension, which play an important role in the pathophysiology of BPD.

## Conclusion

As point of care ultrasound interests, training, and applications grow in the field of neonatology, this study shows promise for the use of lung ultrasound as a non-invasive and reproducible tool to risk-stratify infants with evolving BPD. How lung ultrasound can be used in future diagnostic applications, clarifying responses to existing or novel therapies, and gaining insights into mechanisms of altered respiratory mechanics remains to be studied and seen.

## Data Availability

Request for data sharing should be submitted to the corresponding author for consideration.
